# Progress in Otology

**Published:** 1903-05-02

**Authors:** 


### PROGRESS IN OTOLOGY.

(*Continued from page* 67.)

**The Intra-cranial Complications of Purulent Otitis.** Frederick Eve [10] recently gave a clinical lecture on this subject. The slight character of the symptoms of extradural abscess was remarked upon. Probably this is explainable by the partition between the dilated antrum and the sigmoid groove being partially absorbed so that the pus is not under much pressure. If this partition is intact the symptoms are more distinct and comprise those of acute mastoiditis—local pain, hemicrania referred to the posterior half of the side of the cranium, local œdema, and moderate pyrexia. There may be in addition vomiting, giddiness and mental confusion. In discussing sinus thrombosis the author points out that the aim should be to recognise the disease before the clot begins to disintegrate, and alludes to four cases in which he accomplished this. In none of these had there been a rigor, and the symptoms were mainly referable to the existence of suppuration around the sinus. In three of the cases vomiting was conspicuous. In two there was drowsiness and in two incontinence of urine. It is recommended therefore that the sinus be explored in any case in which there is pyrexia with vomiting, drowsiness, or incontinence of urine. A case of posterior basal meningitis is reported and one of serous meningitis, both secondary to ear disease. Barlow and Lees have shown that in posterior basal meningitis the infection reaches the brain in the majority of cases by means of the ear, and they suggested that in all cases the membrana tympani should be tapped in the early stage of the disease. Mastoidectomy should be performed if purulent otorrhœa be present. Relief has been obtained by tapping the lateral ventricle. Discussing cerebellar abscess the author alludes to McBride's sign. McBride suggested that in these cases the septic organisms travelled from the *internal* ear to the posterior surface of the petrous bone and thence to the cerebellum, and that the labyrinth being destroyed a tuning fork would not be heard on the affected side. But the infection appears sometimes to travel by means of the sigmoid sinus and thence to the cerebellum. McBride's sign is therefore only of value when bone conduction is absent.

**Malignant Disease of the Ear.**—Edward B. Dench[11] recently reported a case of malignant disease of the middle ear involving the cranial cavity. The patient, a child eighteen months old, was brought to the clinic with a history of watery discharge from the ear of 10 days' duration, of restlessness and of the development of a squint. The last condition was due to paralysis of the right external rectus muscle. The canal of the ear was found to be occupied by a tumour apparently covered with a thin layer of epithelium. A mastoid operation revealed a cauliflower-like growth in the cortex over the antrum and tympanum, involving the cranial cavity and extending nearly to the apex of the petrous bone. The mass and the softened bone around it were removed, and the growth afterwards found to be an endothelial sarcoma. In a few weeks there were evidences of recurrence, and the question arose whether more should be done. Dench proposed to make another attempt to eradicate the growth and at the same time to ligate the carotids in order to retard and prevent recurrence. Dench also alluded to three cases he had had of malignant growth of the auricle. In one all the glands in the corresponding side of the neck were involved, and had been removed with the growth. The patient was alive and well four years later. In another case, one of melano-sarcoma, the operation was followed by slight recurrence, but after a second operation there had been complete relief. The results of operation in such cases were said to be exceedingly good.

[10] Lancet, Jan. 17, 1903, p. 149. [11] Med. Rec., Nov. 1, 1902.

